# Quality Detection for Dragon Fruit Based on the End-of-Arm Spectral Sensor of the Harvesting Robot

**DOI:** 10.3390/foods15111944

**Published:** 2026-06-01

**Authors:** Zongxiu Bai, Qiu Xu, Kairan Lou, Bin Zhang

**Affiliations:** 1 School of Mechanical and Electrical Engineering, Hainan University, Haikou 570228, China; 996909@hainanu.edu.cn (Z.B.); 23220855000012@hainanu.edu.cn (Q.X.); 22210828010001@hainanu.edu.cn (K.L.); 2 School of Food and Science Engineering, Hainan University, Haikou 570228, China

**Keywords:** spectral sensor, dragon fruits, machine learning, detection, harvesting robot

## Abstract

Carrying out quality grading detection on the harvested dragon fruit is an important step in the dragon fruit industry. To reduce the high costs and damage rates caused by this process, an online spectral sensor and a weighing sensor embedded at the end effector of the dragon fruit-picking robot were designed to detect the sugar content, hardness and weight of the dragon fruits in real time during the picking process, thereby achieving the quality classification of the dragon fruits. After collecting the spectral data of dragon fruit, typical linear and nonlinear machine learning methods were used to establish prediction models for SSC-edge, SSC-center and hardness of dragon fruit. The results showed that PLSR models were selected as optimal models for prediction sugar content and hardness, and *R*^2^ of test set for SSC-edge, SSC-center and hardness are 0.876, 0.826 and 0.902, respectively. Subsequently, the dragon fruits were classified based on the weighing sensor, and the SSC-center and hardness were predicted. The results showed that the established quality prediction model and the prototype could achieve the integrated operation of non-destructive quality detection and grading of dragon fruit during picking. The study provides technical support for the intelligent upgrade of fruit-harvesting equipment and the grading operations.

## 1. Introduction

As a typical tropical and subtropical fruit, dragon fruit has seen a growing market demand due to its rich nutritional value and unique taste. As the consumption scale of fruits expands, consumers are paying more attention to the taste, quality and nutritional value of fruits, which further drives the market demand for high-quality fruits [1]. At present, the harvesting of dragon fruit is still mainly carried out manually. During the harvesting process, the fruit cannot be sorted simultaneously. After being transported to the processing workshop, subsequent sorting operations need to be carried out by manual labor or specialized sorting equipment. The process increases the labor intensity of fruit farmers and is prone to causing secondary damage to the fruits. Therefore, developing integrated equipment for dragon fruit picking and grading is an inevitable trend for the healthy development of the dragon fruit industry.

The traditional quality inspection of dragon fruit relies on manual inspection methods, where the fruit is classified based on its size, color, texture and maturity. This method is highly subjective, prone to errors, and lacks scalability for large-scale production [2]. With the development of sensor technology and artificial intelligence, non-destructive detection techniques such as flexible tactile sensing arrays [3], machine vision [4,5], electronic nose detection [6,7,8], hyperspectral imaging [9,10,11] and spectroscopy [12,13,14], combined with machine learning methods, have gradually been applied to the quality inspection of fruit, driving the dragon fruit industry to shift from relying on experience to being driven by digital intelligence. Although electronic noses and hyperspectral technology have great potential for application in fruit detection, they enable non-destructive inspection of fruits. The high cost of these devices, the low efficiency in data processing and modeling, and large amount of hardware and storage space, make them unsuitable for robot end-point detection.

Recently, multispectral sensors have made significant progress in the field of fruit quality detection due to the low cost and portability [15]. In 2022, Zhang et al. [16] developed a prototype of a portable visible light/near-infrared analysis device based on multispectral sensors, which is used for quickly identifying the ripeness of apples. In 2024, Zhu et al. [17] developed a handheld device for non-destructive detection of soluble solids in butter peaches using visible near-infrared spectroscopy technology. In 2026, Guo et al. [18] using near-infrared spectroscopy with flexible transmission tray and deep learning, achieved online detection of apple moldy core. Above studies have applied spectral technology to the online detection of fruit quality, but most of them are conducted in static laboratory environments. Under the trend of large-scale orchard cultivation and intelligent management, the application of harvesting robots has become a key technical path to reduce labor costs and improve operational efficiency. However, the current picking robots mainly rely on machine vision to identify and locate the fruits, and then complete the picking operation. They are unable to make real-time judgments on the internal quality of the fruits (such as sugar content, hardness, and weight) during the picking process. It led to the separation of the picking process from the subsequent sorting stage, which not only increased logistics costs but also failed to meet demand for traceability of high-quality fruits. Therefore, developing a device that can immediately sense the quality of fruits during the picking process and achieve integration of picking and inspection has significant academic value and industrial application prospects.

Integrating spectral detection technology with the end effector can significantly contribute to enhancing the intelligence level of agricultural production. Peng et al. [19] integrated optical sensors and gripping mechanisms at the end of a six-axis robotic arm, constructing an automatic apple internal quality detection and grading robot system, which achieved integration of apple grasping, spectral acquisition, sugar content detection and grading operations. Cortés et al. [20] developed a robotic gripper that integrates tactile sensing and visible-near-infrared reflectance spectroscopy detection, which could simultaneously obtain mechanical and spectral information during the mango handling process, thereby evaluating the fruit maturity. Blanes et al. [21] designed a robot gripper equipped with an acceleration sensor to conduct non-destructive assessment of hardness, soluble solid content, and maturity index during the process of mango grasping and transportation. The above studies verified the feasibility of conducting fruit harvesting and quality inspection simultaneously, providing a reference for the integrated design of spectral sensing and end effectors. However, both the detection system and the end effectors generally have the problem of large size, making them difficult to adapt to orchards and complex mobile operation scenarios. It is necessary to further enhance the high integration of the end effectors and the detection system and reduce the overall size to adapt to the picking scenarios. Qiu et al. [22] integrated a reflective near-infrared maturity sensor and an endoscope camera into a miniaturized end-effector structure, achieving the integration of flexible grasping and maturity identification of blackberries. Liu et al. [23] developed a hand-like gripper embedded with flexible gel sensor for tomato harvesting, which had a soft touch and could intelligently sense the maturity of the fruits. Ge et al. [24] proposed multi-view gripper internal sensing for the regression of strawberry ripeness using a mini-convolutional neural network for robotic harvesting. Dai et al. [25] placed the spectral detection module outside the robot fingers, enabling it to identify the maturity, hardness and sugar content of the fruits during the grasping process, thereby enhancing the mobility flexibility of the end effector. In conclusion, the current integration of spectral detection systems with end effectors still faces issues such as high cost and insufficient system integration. Therefore, it is urgent to develop a portable, low-cost, highly integrated end-effector-integrated spectral detection system, providing key technical support for agricultural robots to achieve intelligent operations. Furthermore, the existing research on dragon fruit-picking robots mainly focuses on their picking characteristics [26], target recognition and detection [27,28,29], path planning [30], and robot control systems [31], but lacks studies on the collection of quality grading during the picking process.

In conclusion, a method for detecting the quality of dragon fruit based on the end-of-arm spectral sensor of the picking robot was proposed to meet the integrated operation requirements of dragon fruit harvesting and quality grading. Firstly, the spectral sensor and the weighing sensor were integrated into the end effector of the harvesting robot, and a fire fruit-grading detection system based on the harvesting robot was developed. Secondly, a dragon fruit quality index detection model based on typical machine learning was constructed, and the prediction models for SSC and hardness of dragon fruit were optimized. Finally, the accuracy and real-time performance of this system in grading the sugar content and identifying internal defects of fire fruit during dynamic picking were verified through picking experiments. The study results provide theoretical basis and technical support for the harvesting and grading of dragon fruit and promote the healthy development of the dragon fruit industry.

## 2. Materials and Methods

### 2.1. Design of Spectral Detection and Weighing System

#### 2.1.1. Hardware System Integration

**Figure 1:**
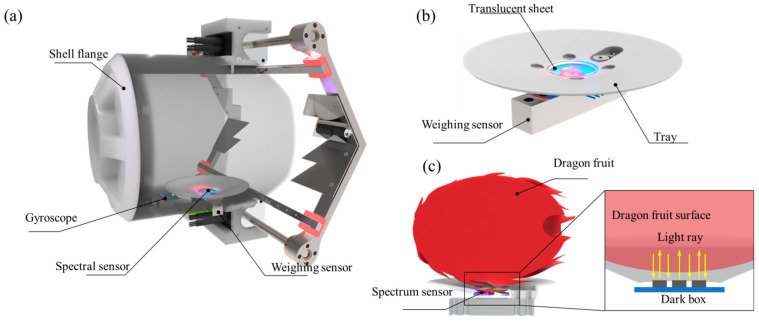
Composition of the spectral detection structure: (**a**) Internal structure diagram of the end effector. (**b**) Structure of the spectral and weighing module. (**c**) Schematic diagram of the dark box during the spectral data collection of dragon fruit.

A spectral sensor was embedded at the end of the harvesting mechanical arm in this study, and an online quality detection system for dragon fruit during the harvesting period was established. The system is mainly composed of a spectral sensor and a weighing sensor as its core components (Figure 1). Spectral sensors are used to characterize the internal quality parameters of dragon fruit, while weighing sensors enable precise measurement of the weight of the fruit. Among them, the spectral sensor adopts the AS7265X developed by AMS OSRAM Company (Graz, Austria). It is composed of three sensors, namely AS72651, AS72652, and AS72653. The sensor is small in size, simple in structure, and has stable detection performance. The AS7265X circuit board integrates surface-mount LEDs, and the detectable spectral range is from 410 to 940 nm, with a total of 18 channels. To reduce the influence of sunlight or ambient light during the picking and inspection process, the spectral sensor is placed inside the harvesting cylinder. The spectral sensor is installed and fixed beneath the tray. The core spectral detection structure of this device is placed beneath the tray, and the concave surface of the fruit is closely attached to the surface of the tray to form a dark chamber.. The sensor hole in the middle of the tray, with a certain degree of concavity on the surface, enables the cut dragon fruit to fit the surface of the tray and fix the distance between the spectral sensor and the surface of the dragon fruit. The tray and the surface of the dragon fruit are closely adhered to form a closed local dark field, effectively physically blocking external stray light and weakening the interference of light changes on the spectral data, achieving stable dark box detection results (Figure 1c). The bottom of the tray is connected to the weighing sensor, and the entire component is fixed inside the harvesting drum. The weighing sensor is a cantilever beam inductive sensor with a measurement range of 2 kg. Its parameters are shown in Table 1. To reduce the detection error of the weighing sensor, an attitude sensor was horizontally fixedly installed inside the harvesting cylinder. This sensor could monitor the working posture of the end effector in real time, and achieve compensation for and correction of the weighing error through the dynamic feedback of the attitude information.

#### 2.1.2. Development of Upper-Level Machine Software

**Figure 2:**
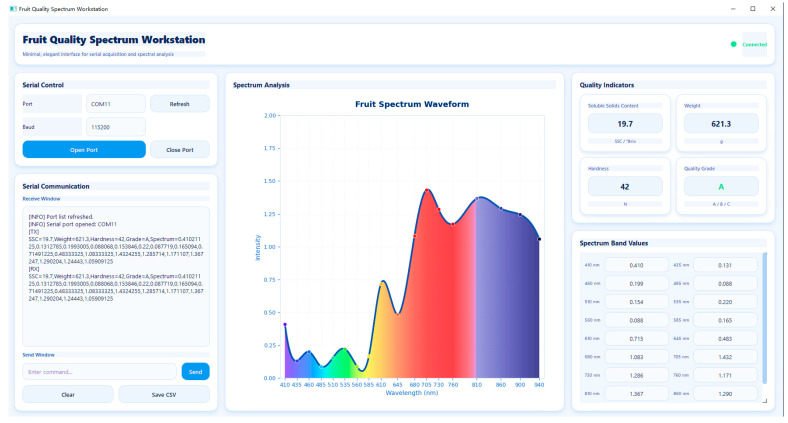
The interface of the spectral data acquisition software.

To achieve real-time acquisition, display, analysis and storage of multi-band spectral information of fruits, the spectral data acquisition software for dragon fruit was developed based on PyQt5. The software (Figure 2) is mainly used for serial communication with the lower-level machine spectral acquisition module, and it can achieve functions such as receiving spectral data, visualizing waveforms, displaying quality parameters, and archiving data. The software interface is designed in a modular way, taking into account both ease of operation and visual appeal. It can meet the requirements for fruit quality inspection and data management in laboratory environments. The upper-level machine software was developed using the Python language. The PyQt5 was used as the graphical interface framework, PySerial was combined to achieve serial communication, and Matplotlib 3.8.2 was integrated to draw spectral curves. The software is mainly composed of a serial communication module, a data parsing module, a spectrum display module, a quality parameter display module, and a data storage module. Among them, the serial communication module is responsible for serial port identification, baud rate setting, serial port opening and closing, and data sending and receiving. The data parsing module is responsible for extracting fields from and converting formats of the received string data. The spectral display module is responsible for plotting the reflectance or response values of 18 bands as spectral curves and performing spline fitting and smooth filling display. The quality parameter display module is responsible for updating the results such as SSC, weight, hardness and quality grade in real time on the interface. The data storage module is responsible for saving the collected results as CSV files, so that they can be used for subsequent statistical analysis and model training.

### 2.2. Collection of Spectral Data for Dragon Fruit and Determination of Quality Indicators

**Figure 3:**
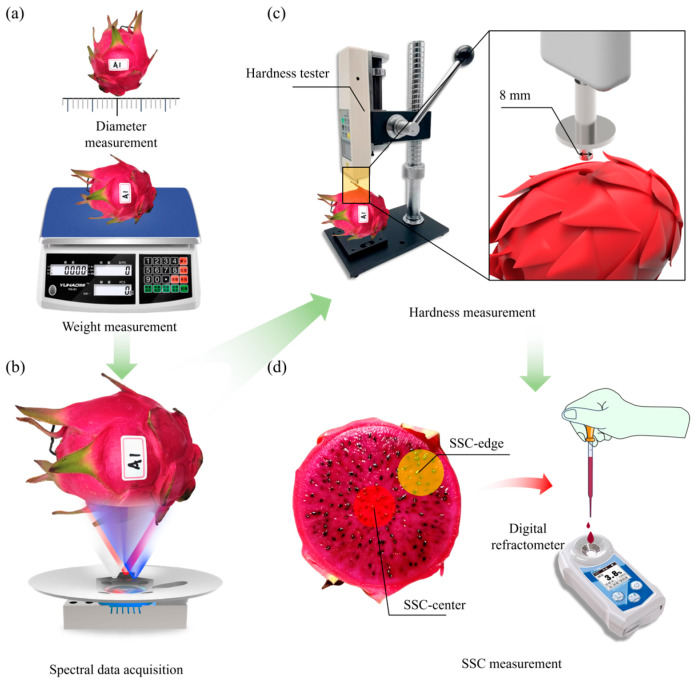
Flowchart of the test process for sugar content, weight and hardness of dragon fruit: (**a**) Measurement of fruit weight and diameter. (**b**) Spectral detection. (**c**) Hardness test. (**d**) Sugar content test.

For this study, 200 “Jindu Yihao” dragon fruit samples that were picked on the same day at the dragon fruit planting base in Le Dong City, Hainan Province, China (geographic coordinates: 18°24′–18°58′ N, 108°39′–109°24′ E), were selected as the experimental materials. The samples included fruits of various sizes and different quality levels, and the relevant experiments were conducted promptly after refrigerated transportation. All the fruits were given unique identification numbers and then placed in a 22 °C constant-temperature laboratory environment to complete all the experimental procedures. The sample data collection process is shown in Figure 3.

In this study, the spectral data and weighing data of the samples were collected simultaneously. The end effector was adjusted to a horizontal position, and the spectral sensor was preheated. After the preheating was completed, spectral data of the equatorial part of the dragon fruit samples were collected. Each fruit was measured five times, and the average value was taken as the final spectral measurement value of the fruit. At the same time, the sample weight data were measured and recorded simultaneously, serving as the output value of the end weighing sensor in the actual detection state. Each fruit was measured three times, and the average value was taken as the weighing sensor measurement result of the sample.

After the sensor data collection was completed, the actual weight of each fruit was measured using an electronic scale, which was taken as the true value of the sample weight. The test data were then entered according to the corresponding numbers. Then, the hardness of the dragon fruits was measured using the GY-4 fruit hardness tester (Adoptech Instruments Co., Ltd., Leqing, China), which was equipped with an 8 mm diameter probe. During the measurement, a test point was selected at the equatorial part of the fruit. The probe was placed perpendicular to the surface of the fruit and slowly and uniformly inserted into the flesh until the scale line of the probe was level with the surface of the fruit. The maximum pressure during the measurement process was selected as the hardness characteristic value of the fruit. The SSC of the fruits was determined using the AK002B high-precision digital refractometer (AIOK, Shenzhen Side Technology Co., Ltd., Shenzhen, China). The measuring range of this instrument is 0 to 45%, and the measurement accuracy is up to 0.2%. After the hardness measurement was completed, the dragon fruit was cut open. The fruit flesh from the center and the edge was extracted and juiced separately. The impurities were filtered out, and the juice was dripped onto the detection area of the sugar meter for measurement. The detection area was washed with distilled water and dried when each measurement was completed. Each sample was measured three times in repetition, and the average value is taken as the final SSC measurement result of the fruit.

### 2.3. Data Preprocessing

#### 2.3.1. Spectral Data Correction

**Figure 4:**
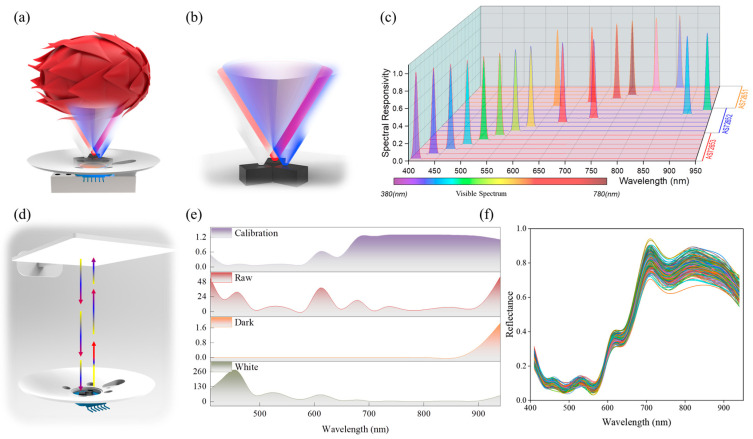
Spectral band channels and sensor calibration: (**a**) schematic diagram of spectral sensor detection; (**b**) positions of the three spectral sensors; (**c**) corresponding spectral band ranges of the three spectral sensors; (**d**) schematic diagram of spectral sensor calibration; (**e**) calibrated spectral curves; (**f**) spectral curve of dragon fruit after calibration.

The process of collecting and correcting the spectral data of the dragon fruit samples is shown in Figure 4. The spectral sensor needs to be calibrated before use. It is calibrated using the diffuse reflection principle by means of a white light panel. The calculation is carried out as follows:

(1) $$R_{cal}=\frac{R_{raw}-R_{dark}}{R_{white}-R_{dark}}\times 100\%$$

Among them, *R_cal_* represents the corrected spectral value, *R_raw_* represents the original spectral value, *R_dark_* represents the dark spectrum, and *R_white_* represents the white spectrum.

To improve the accuracy of the prediction model for soluble solids content (SSC) and hardness of dragon fruit, this study performed maximum–minimum normalization on the spectral reflectance data. This method linearly maps the original data to the interval [0, 1], effectively eliminating the differences in scale and enhancing the model’s sensitivity to spectral features. At the same time, the spectral curves were fitted and represented by B-spline curves. The 200 processed dragon fruit spectral curves are shown in Figure 4f.

The average spectral curves of 200 samples are shown in Figure 4f. The spectral change trend is basically consistent with the detection results of Luo Xia et al. [9]. The spectral curve of the whole dragon fruit shows distinct-phased changes in the visible-near-infrared wavelength range. In the visible light region, the curve is generally low with local absorption fluctuations, mainly related to the selective absorption of betacyanin, chlorophyll and other pigment components in the fruit peel. As the wavelength increases, the curve rises rapidly near the red edge, indicating that the scattering effect of light by the fruit skin and underlying tissues becomes stronger. After entering the near-infrared region, the spectral reflectance remains at a relatively high level and shows a certain degree of slow fluctuation. This is mainly related to the internal tissue structure of the fruit and the absorption characteristics of chemical bonds such as O-H and C-H in water and organic substances. If there is a downward trend near 970 nm, it indicates that this wavelength band is significantly affected by water absorption. This suggests that the sensor designed for this study of dragon fruit can be used to obtain the spectral data of dragon fruit.

#### 2.3.2. Calibration and Filtering of Weighing Sensors

**Figure 5:**
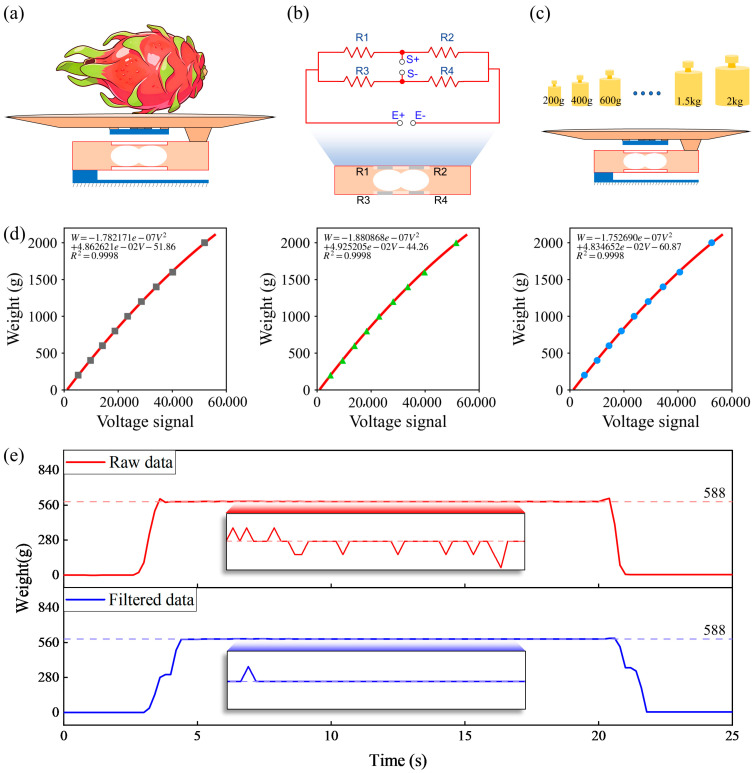
Calibration and filtering of load sensors: (**a**) Cantilever beam type load sensor. (**b**) Wheatstone bridge circuit. (**c**) Calibration of load sensor. (**d**) Secondary fitting curve for weight calibration. (**e**) Comparison of filtering effects.

The weighing sensor designed in this paper is shown in Figure 5a. The four strain resistors attached to the surface of the elastic body in the weighing sensor respectively form a Wheatstone full-bridge circuit, which are denoted as R1, R2, R3 and R4. The excitation ends of the bridge circuit are E+ and E−, and the differential signal output ends are S+ and S− (as shown in Figure 5b). When an external load acts on the elastic body, the resistance of each arm of the bridge circuit undergoes a slight change, thereby outputting a voltage signal proportional to the load. The voltage signal is amplified and converted into digital form by the HX711 module, converting the analog output of the sensor into a digital signal and transmitting it to the microcontroller system. Finally, the microcontroller processes and interprets the collected digital signals, and converts them into corresponding weight values based on the pre-established calibration relationship.

To obtain the quantitative relationship between the voltage signal and the actual weight, the weighing system was subjected to static calibration. As shown in Figure 5c, standard weights were successively placed at the center of the weighing tray, with the loading masses set at 200, 400, 600 g, up to 2000 g. After each load level was stabilized, the output voltage signal of the sensor was recorded, and the measurement was repeated three times to reduce random errors. Based on the calibration experimental data, a quadratic linear fitting with the standard weight was performed as the dependent variable and the voltage signal was output as the independent variable to establish a weight conversion model. As can be seen from Figure 5d, within the test range, there is a good linear relationship between the weight and the voltage signal. The coefficient of determination R^2^ is close to 0.9998, indicating that this device has a high degree of linearity and calibration accuracy. The three sets of calibration results all show a high degree of consistency, indicating that the weighing system has good repeatability and stability. For scenarios such as fire fruit picking operations and field-level online measurement, where the detection data are prone to contain random noise and abnormal burrs, the weighing sensor employs the median mean filtering algorithm to perform real-time filtering on the detection data. In this study, the filtering window was set to five, and the real weight of 588 g of dragon fruit was tested. The filtering effect is shown in Figure 5e. Compared with a single filtering algorithm, the median mean filtering cannot only precisely suppress the random noise and sudden abnormal jumps in the weighing signal but can also maximize the retention of the true change trend of the data. It has the advantages of excellent noise reduction effect, short response delay, and strong real-time performance, and can effectively improve the stability and measurement accuracy of the weighing detection data, meeting the engineering requirements for real-time monitoring of dynamic loads in agricultural scenarios.

### 2.4. Model Establishment and Evaluation

To achieve the prediction of the SSC and hardness of dragon fruit, all the samples were divided into a validation set and a prediction set in a ratio of 7:3. Among them, the number of samples in the validation set was 140, and the number of samples in the prediction set was 60.

The main task of this research was to develop an online spectral system for detecting the quality of dragon fruit based on the end effector of the picking robot. Considering the computational cost and the feasibility of deployment, the classic machine learning algorithms of Multiple Linear Regression (MLR), Partial Least Squares Regression (PLSR), Support Vector Regression (SVR), Extreme Learning Machine Regression (ELMR), Generalized Regression Neural Network (GRNN), K-Nearest Neighbors (KNN), Back Propagation Neural Network (BPNN), Random Forest (RF), eXtreme Gradient Boosting (XGBoost) were selected to respectively model and analyze the central sugar content (SSC-center), edge sugar content (SSC-edge) and hardness of dragon fruit, and to conduct a comparative analysis of the prediction performance of different models.

To comprehensively evaluate the performance of the established model on both the training set and the prediction set, root mean square error and correlation coefficient (*RMSE*) and correlation coefficient (*R*^2^) were selected as the main evaluation indicators. The calculation formulas are as follows.

(2) $$RMSE=\sqrt{\frac{1}{N}{\sum_{i=1}^{N}\left(Y_{i}-\hat{Y_{i}}\right)}^{2}}$$

(3) $$R^{2}=\frac{\sum_{i=1}^{N}{\left(Y_{i}-\hat{Y}\right)}^{2}}{\sum_{i=1}^{N}{\left(Y_{i}-\bar{Y}\right)}^{2}}$$

where $Y_{i}$ and $\hat{Y_{i}}$ represents the predicted value and measured value, *N* is the number of samples, and $\bar{Y}$ is the average value.

The value of *R*^2^ ranges from 0 to 1. The larger the *R*^2^ value, the higher the correlation between the predicted values and the measured values. *R*^2^ < 0.82 indicates poor model performance, 0.82 ≤ *R*^2^ ≤ 0.90 indicates good model performance, and *R*^2^ > 0.90, it indicates excellent model performance. *RMSE* reflects the degree of fit between the actual values and the model established. The smaller the RMSE means the better the model prediction.

## 3. Results and Discussion

### 3.1. Modeling Results and Analysis of SSC-Center for Dragon Fruit

**Figure 6:**
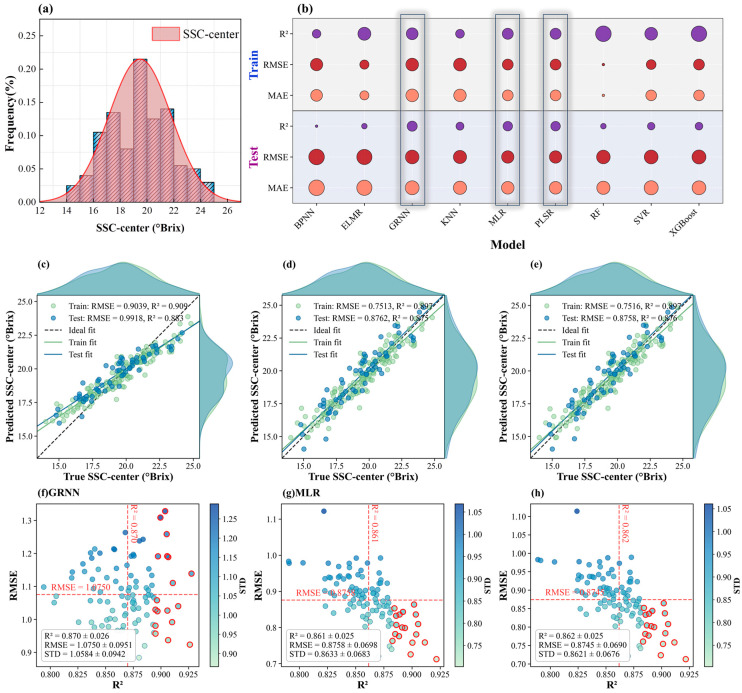
Prediction results of the sugar content at the center for the dragon fruit. (**a**) The data distribution of the SSC-center of the sample. (**b**) Comparison results of the modeling outcomes of 9 algorithms. Model prediction result graph: (**c**) GRNN. (**d**) MLR. (**e**) PLSR. Model stability test results: (**f**) GRNN. (**g**) MLR. (**h**) PLSR.

The SSC-center data of the dragon fruit samples were analyzed, and nine classic machine learning methods were used to predict the central sugar content of dragon fruit. The results are shown in Figure 6.

As is shown in Figure 6a, the SSC-center of the dragon fruit samples generally follows a nearly normal distribution, indicating that the sample distribution is relatively continuous and there is no obvious abnormal skewness. It could be used for subsequent modeling analysis. A comprehensive comparison of the modeling results of 9 algorithms was conducted, and the results are shown in Figure 6b. If there is a significant performance disparity between the training set and the test set of the model, indicating that the model is overfitting. From Figure 6b, it can be seen that the *R*^2^ of the GRNN, MLR, and PLSR models are relatively high, and the performance differences between the training set and the test set are small, indicating that there is no overfitting phenomenon in the models. Therefore, further analysis of the results of these three models is shown in Figure 6c–e. As can be seen from Figure 6c, the GRNN model demonstrates strong fitting ability on the training set, with *R*^2^ and *RMSE* on the training set is 0.909 and 0.9039, respectively, and the *R*^2^ and *RMSE* on the test set is 0.883 and 0.9918, respectively. It indicated that the GRNN could effectively capture the nonlinear relationship between multispectral information and SSC-center, and the model had high fitting accuracy. However, compared with the other two models, the performance gap between the training set and the test set of the GRNN was relatively larger. In particular, the *RMSE* of the test set was larger, indicating that while it had a stronger fitting ability, its generalization stability was slightly inferior to the MLR and PLSR models. As shown in Figure 6d, the *R*^2^ and *RMSE* of the training set for the MLR model is 0.897 and 0.7513, respectively; the *R*^2^ and *RMSE* of the test set is 0.875 and 0.8762, respectively. Overall, the fitting results of the training set and the test set were quite similar. Most of the scattered points were located near the ideal fitting line, indicating that MLR model had good generalization ability and consistent prediction performance. Although the *R*^2^ of MLR on the training set was slightly lower than that of GRNN, its error on the test set was smaller, indicating that the model structure was simpler and had a stronger ability to resist overfitting. As shown in Figure 6e, the *R*^2^ and *RMSE* of the training set for the PLSR model is 0.897 and 0.7516, respectively; the *R*^2^ and *RMSE* of the test set is 0.876 and 0.8758, respectively. As shown in Figure 6e, the *R*^2^ and *RMSE* of the training set for the PLSR model is 0.897 and 0.7516, respectively; the *R*^2^ and *RMSE* of the test set is 0.876 and 0.8758, respectively. The result was very close to the MLR model, but the *R*^2^ of the test set was slightly higher and the *RMSE* was slightly lower. It indicated that PLSR maintained a strong explanatory ability while having better handling capabilities for the collinearity problem among multispectral variables. Therefore, it exhibited superior comprehensive performance in the prediction of SSC-center.

Furthermore, the three models were randomly repeated for 100 rounds, and the stability of the models was analyzed. The results are shown in Figure 6f–h. The average *R*^2^ of the 100-round results of the GRNN model is 0.870 ± 0.026, and the average *RMSE* is 1.0750 ± 0.0951. Although its average *R*^2^ was relatively high, the scatter distribution was relatively scattered and the fluctuation range was large. It indicated that the GRNN was relatively sensitive to sample division and had relatively weak stability when repeated modeling was conducted. In contrast, the average *R*^2^ of the MLR model was 0.861 ± 0.025, and the average *RMSE* was 0.8758 ± 0.0698; the average *R*^2^ of the PLSR model was 0.862 ± 0.025, and the average *RMSE* was 0.8745 ± 0.0690. The two sets of data were more concentrated in their distribution. The samples near the mean value indicated by the red dotted line were clearly clustered, indicating that the model had good repeatability and more stable results. It was worth noting that PLSR and MLR exhibited similar prediction accuracy and visualization characteristics in the SSC-center modeling. It was mainly because both of them essentially belong to linear modeling methods. When the relationship between the spectral variables and the target variable was mainly linear, the two models tended to extract similar dominant information. Although PLSR reduced the influence of collinearity among variables by extracting latent variables, under the current data conditions, the main latent variables extracted by PLSR had a high degree of consistency with the effective information utilized by MLR. Therefore, both methods showed similar performance in terms of scatter distribution, fitting trend, and stability. Ultimately, after comprehensive consideration of factors such as prediction accuracy, anti-overfitting ability, and model stability, PLSR was selected as the optimal model for multispectral prediction of the SSC-center of dragon fruit.

### 3.2. Modeling Results and Analysis of SSC-Edge for Dragon Fruit

**Figure 7:**
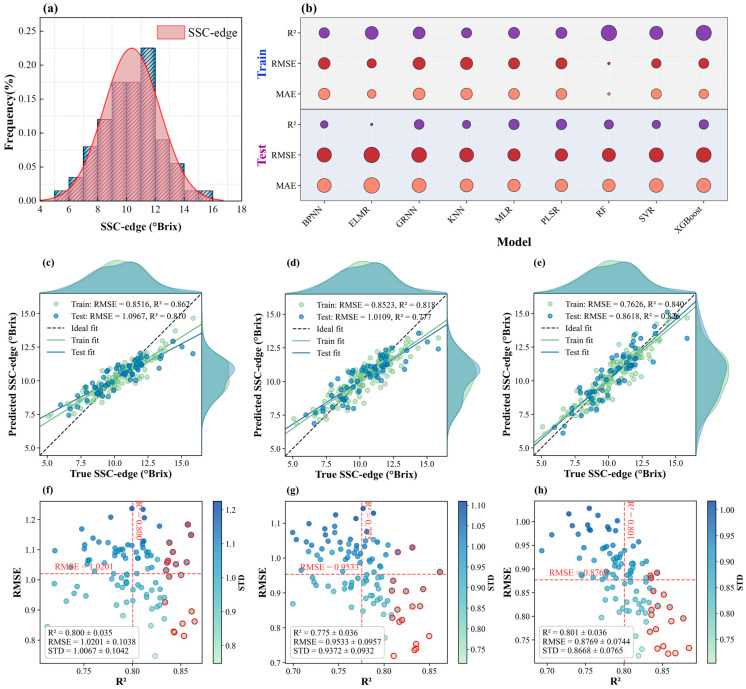
Prediction results of the sugar content at the edge for the dragon fruit. (**a**) The data distribution of the SSC-edge of the sample. (**b**) Comparison results of the modeling outcomes of 9 algorithms. Model prediction result graph: (**c**) GRNN. (**d**) KNN. (**e**) PLSR. Model stability test results: (**f**) GRNN. (**g**) KNN. (**h**) PLSR.

The SSC-edge data of the dragon fruit samples were analyzed, and 9 classic machine learning methods were used to predict the central sugar content of dragon fruit. The results are shown in Figure 7.

As is shown in Figure 7a, the SSC-edge of the dragon fruit samples generally follows a nearly normal distribution, indicating that the sample distribution is relatively continuous and there is no obvious abnormal skewness. The comparison of the results of the 9 algorithms is shown in Figure 7b. By comprehensively comparing *R*^2^ and RMSE, it was found that the GRNN, KNN and PLSR models performed the best. Further analysis on their modeling results was carried out, and the results were shown in Figure 7c–e. As shown in Figure 7c, the *R*^2^ and *RMSE* of the training set for the GRNN model is 0.862 and 0.8516, respectively; the *R*^2^ and *RMSE* of the test set is 0.870 and 1.0967, respectively. It indicated that the model had a good nonlinear fitting ability, but the error of the test set was relatively large, and there was still room for improving the stability of the prediction. As shown in Figure 7d, the *R*^2^ and *RMSE* of the training set for the KNN model is 0.818 and 0.8523, respectively; the *R*^2^ and *RMSE* of the test set is 0.767 and 1.0109, respectively. Compared with GRNN and PLSR, the *R*^2^ of the test set for the KNN model had decreased significantly, while *RMSE* was relatively high. It indicated that its generalization ability was relatively weak. As shown in Figure 7e, the *R*^2^ and *RMSE* of the training set for the PLSR model is 0.840 and 0.7626, respectively; the *R*^2^ and *RMSE* of the test set is 0.826 and 0.8618, respectively. In comparison, PLSR has a higher *R*^2^ and lower *RMSE* on the test set, indicating that its prediction accuracy and generalization ability were better.

Three preferred models were subjected to 100 repetitions of modeling, and the results are shown in Figure 7f–h. The average *R*^2^ of the GRNN model was 0.800 ± 0.035, and the average *RMSE* was 1.0201 ± 0.1038. The results showed a relatively large degree of dispersion, indicating that it was sensitive to sample division and had relatively weak stability. The average *R*^2^ of the KNN model was 0.775 ± 0.036, and the average *RMSE* was 0.9533 ± 0.0957. Overall, its performance was inferior to that of GRNN and PLSR, and the results of repeated modeling showed significant fluctuations. The average *R*^2^ of the PLSR model was 0.801 ± 0.036, and the average *RMSE* was 0.8769 ± 0.0744. The scatter plot distribution was more concentrated and the fluctuation range was smaller, indicating that the model had good repeatability and stability. Overall, GRNN had a strong fitting ability, but the error and fluctuation were relatively large; the KNN model had a relatively weak prediction effect and insufficient generalization ability; PLSR performed best in terms of prediction accuracy, generalization ability and stability, and therefore could be used as the optimal model for predicting the SSC-edge of dragon fruit using multispectral data.

### 3.3. Modeling Results and Analysis of Hardness for Dragon Fruit

**Figure 8:**
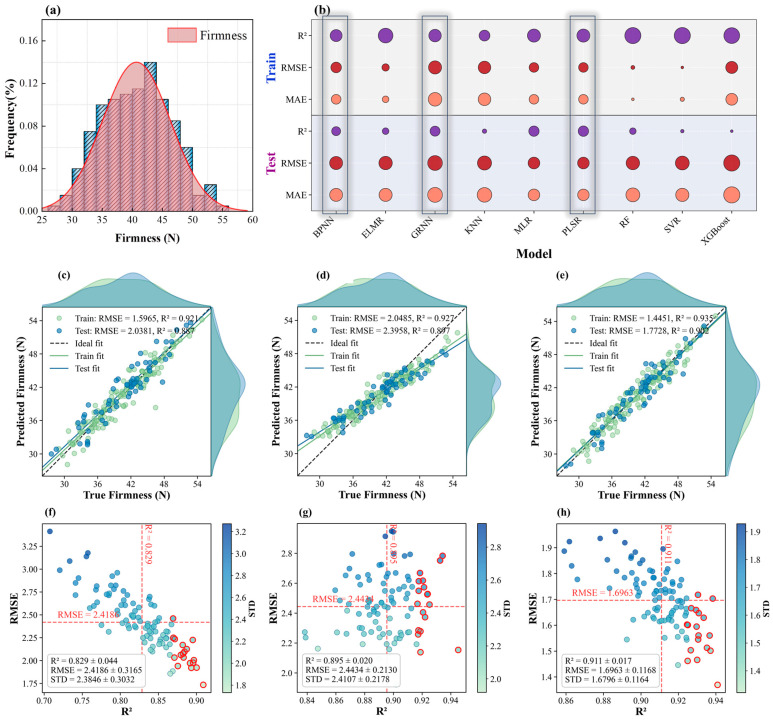
Prediction results of hardness for the dragon fruit. (**a**) The data distribution of the hardness of the sample. (**b**) Comparison results of the modeling outcomes of 9 algorithms. Model prediction result graph: (**c**) BPNN. (**d**) GRNN (**e**) PLSR. Model stability test results: (**f**) BPNN. (**g**) GRNN. (**h**) PLSR.

The hardness data of the dragon fruit samples were analyzed, and 9 classic machine learning methods were used to predict the central sugar content of dragon fruit. The results are shown in Figure 8.

After comprehensive comparison, the BPNN, GRNN and PLSR models performed the best. As can be seen from Figure 8c, the *R*^2^ and *RMSE* of the training set for the BPNN model is 0.921 and 1.5965 N, respectively; the *R*^2^ and *RMSE* of the test set is 0.887 and 2.0381 N, respectively. It indicated that the model had good fitting ability and prediction performance. As shown in Figure 8d, the *R*^2^ and *RMSE* of the training set for the GRNN model is 0.927 and 2.0485 N, respectively; the *R*^2^ and *RMSE* of the test set is 0.897 and 2.3958 N, respectively. Although its *R*^2^ was relatively high, the *RMSE* of the test set was relatively large, indicating that the prediction error of the model was still relatively high. As shown in Figure 8e, the *R*^2^ and *RMSE* of the training set for the PLSR model is 0.935 and 1.4451 N, respectively; the *R*^2^ and *RMSE* of the test set is 0.902 and 1.7728 N, respectively. Compared with BPNN and GRNN, PLSR had a higher *R*^2^ and lower *RMSE* on the test set, indicating that its prediction accuracy and generalization ability were better.

The 100 repeated models were conducted for the 3 preferred models, and the results are shown in Figure 8f–h. The average *R*^2^ of the BPNN model was 0.829 ± 0.044, and the average *RMSE* was 2.4186 ± 0.3165 N; the average *R*^2^ of the GRNN model was 0.895 ± 0.020, and the average *RMSE* was 2.4434 ± 0.2130 N; the average *R*^2^ of the PLSR model was 0.911 ± 0.017, and the average *RMSE* was 1.6963 ± 0.1168 N. In comparison, the results of the PLSR model were more concentrated and had a smaller fluctuation range, indicating better repeatability and stability. Overall, BPNN, GRNN and PLSR could all be used for predicting the hardness of dragon fruit. BPNN had a better fitting ability, while GRNN showed a higher correlation. However, both of them were inferior to PLSR in terms of error control and stability. PLSR performed best in terms of prediction accuracy, generalization ability and stability. Therefore, PLSR could be regarded as the optimal model for multispectral prediction of the hardness for dragon fruit.

### 3.4. Test of Composite Quality Grading for Dragon Fruit

To verify the accuracy and reliability of the multispectral detection system and the quality prediction model installed on the end effector in the actual picking operation, a complete machine test for the quality grading detection of dragon fruit was conducted. The experiment was conducted on 30 samples of “Jindu No.1” dragon fruit. The end effector was used to simultaneously complete spectral acquisition, weight measurement, posture compensation and quality parameter prediction. The optimal model was utilized to predict the central sugar content and hardness values of the 30 dragon fruits. Subsequently, the weighed validation of the dragon fruits was conducted using an electronic scale, and the SSC and hardness were measured to verify the detection results. To validate the dragon fruits and to achieve automated fruit grading based on the predicted results. The actual measured values and the predicted values were compared to evaluate the performance of the grading system. 30 dragon fruit samples were tested for SSC-center, hardness and overall quality grade.

**Figure 9:**
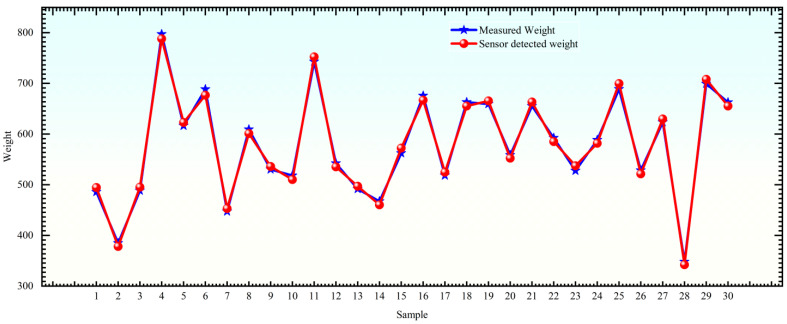
Comparison results of the actual weights of 30 dragon fruit samples and the weights detected by the weighing sensor.

According to the Dragon fruit grade specifications as stipulated in NY/T 3601-2020 [32], dragon fruits can be classified into three grades. In the market, dragon fruits are usually categorized by their size. The prices of different grades of dragon fruits vary significantly. Based on market research and expert experience, red-skinned and red-fleshed dragon fruits with a weight greater than 500 g are classified as top-grade fruits, those weighing between 300 and 500 g are classified as first-grade fruits, and those weighing less than 300 g are classified as second-grade fruits. Therefore, we classified the 30 fruits by weight, and the results are shown in Figure 9.

**Figure 10:**
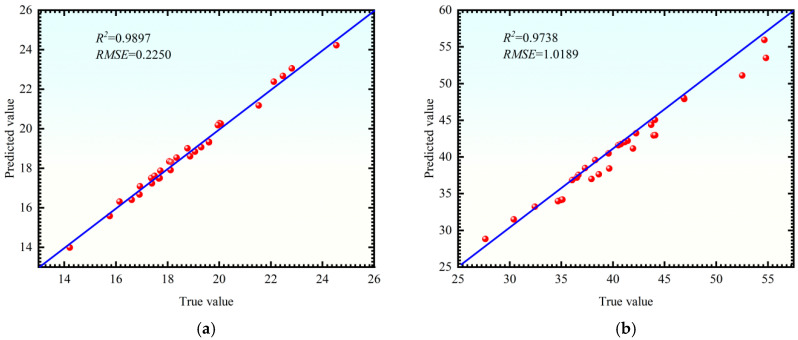
Quality indicator prediction results for 30 dragon fruits. (**a**) SSC-center. (**b**) Hardness.

As shown in Figure 9, the detection values of the weighing sensor are basically consistent with the actual weight change trend. The two curves fit well overall, indicating that the weighing sensor can accurately reflect the actual weight changes in the dragon fruit. From the error indicators in the figure, the average absolute error (*MAE*) of weight detection is 5.46 g, the *RMSE* is 9.24 g, and the average absolute percentage error (*MAPE*) is 1.63%. Among the 30 samples, 21 samples are top-quality fruits and seven samples are first-class fruits. Furthermore, using the optimal model, the SSC-center and hardness of the 30 samples were predicted, and the results are shown in Figure 10.

From Figure 10, it can be seen that by using the optimized PLSR model to predict the SSC-center and hardness of 30 test dragon fruits, good prediction performance was achieved. Among them, the *R*^2^ and *RMSE* for the prediction of SSC-center were 0.9897 and 0.2250, respectively. The *R*^2^ and *RMSE* for the prediction of hardness were 0.9738 and 1.0189, respectively. The model prediction results of both SSC-center and hardness are greater than 0.97, indicating that the model has excellent predictive performance and can provide technical support for in situ non-destructive quality inspection of dragon fruit. Through quality detection and grading during the picking process, certain guarantees can be provided for the subsequent storage and transportation of dragon fruit.

In conclusion, the designed dragon fruit quality detection system can accurately complete the prediction of SSC-center and hardness. Moreover, the weighing detection results are consistent with the actual weight change trend, with a relatively small detection error, which met the application requirements for online quality inspection and grading of dragon fruits at the harvesting end.

## 4. Discussion

At present, most of the research and development efforts for low-cost and portable detection systems are focused on the development of the detection system and the verification of its feasibility. Abasi et al. [33] developed and calibrated a prototype of the instrument using inexpensive parts for apple ripeness detection based on moisture content, soluble solids content, pH, and firmness. The results of this study showed the general ability of this technique for fruit quality inspection. Further work for increasing the accuracy of the instrument and reducing the errors is required. He et al. [34] developed a flexible multimodal optoelectric in situ sensing system that integrates spectroscopy (410–940 nm, 18 channels) and impedance (100 Hz–10 kHz) detection. Using a 0.22 mm-thick flexible substrate, the system achieves conformal contact with the curved surface of mangoes for non-destructive, in situ monitoring. However, practical deployment still faces some limitations, such as Li-ion battery, manual operation, cumbersome in high-density orchards. Liu et al. [35] introduced FEGW-YOLO, a lightweight detection framework explicitly designed to bridge the efficiency-accuracy gap for fine-grained visual perception on edge hardware while maintaining compatibility with multiple sensor modalities. Field deployment in commercial strawberry greenhouses validates an 87.3% harvesting success rate with a 2.1% fruit damage rate, demonstrating feasibility for autonomous systems. It remains an independent on-site detection system and has not yet achieved the simultaneous detection of quality grades during the picking process. Huang et al. [36] introduced a novel multimodal time series graph learning system for mango quality classification, integrating visible/near-infrared imaging and impedance sensing to enable accurate and interpretable harvesting decisions. The researchers proved that future directions include hardware–software co-design for edge deployment and extending the model to support multi-task predictions, such as estimating optimal harvest periods. The above research indicates that there are still certain challenges in deploying low-cost sensors at the harvesting end.

Although this study has demonstrated that embedding spectral sensors and weighing sensors at the end effector of the harvesting robot can achieve dragon fruit grading during the harvesting process, reducing the high costs and damage rates caused by the post-receiving sorting process on the production line, the main focus of this research lies in the design and feasibility testing of the sensor, as well as the data collection and processing which are all conducted indoors, and the testing period is relatively short. However, during the actual harvesting process, the stability of this system still requires further testing. In natural environments, there are significant temperature fluctuations, a lot of dust and high humidity, which have an impact on the stability of the system that needs to be further explored. The endurance capacity of battery is also a key issue that deserves consideration. Our future research will delve into these problems in depth. Despite these challenges, the efficient recognition algorithm and portability of this system have greatly enhanced the efficiency of dragon fruit harvesting and grading, providing solid technical support for precision agriculture.

## 5. Conclusions

An online spectroscopy sensor and a weighing sensor for the end effector of the dragon fruit-picking robot were developed to detect the sugar content, hardness and weight of the dragon fruit fruits in real time during the picking process, and to achieve the quality classification of the dragon fruit. Spectral data of the dragon fruits were collected using spectral sensors. After preprocessing, MLR, PLSR, SVR, ELMR, GRNN, KNN, BPNN, RF and XGBoost machine learning algorithms were utilized to establish and optimize the prediction models for the SSC-center, SSC-edge and hardness of dragon fruit. The results showed that the PLSR models had the best overall performance in predicting the SSC-center, SSC-edge and hardness. The *R*^2^ values of three model test set were 0.876, 0.826, and 0.902, respectively. Further tests on the entire machine were conducted using the end effector, and tests for quality inspection and grading during the dragon fruit-picking process were also carried out. Finally, the weight of the dragon fruit was measured using a weighing sensor. The results showed that it was consistent with the actual weight change trend, with relatively small measurement errors, meeting the requirements for online weighing and grading. The research results showed that the developed end-of-arm spectral sensor combined with PLSR could achieve integrated operations of dragon fruit picking and non-destructive quality detection and grading. The study provides technical support for the intelligence and traceability of the dragon fruit industry.

## Figures and Tables

**Table 1:** The technical parameters of the weighing sensor.

Single-Point Weighing Sensor	Unit	Parameter
Load rating (R.C.)	kg	2
Nominal output (R.O.)	mV/V	1
Composition error	%R.O.	±0.05
Repeatability	%R.O.	±0.03
Creep (30 min)	%R.O.	±0.05
Allowable operating temperature range	°C	−20~+70
Overload (Safe/Ultimate)	%R.C.	150/200
Elastic element material	-	Aluminum alloy

## Data Availability

The original contributions presented in this study are included in the article. Further inquiries can be directed to the corresponding author.
